# Enhancing antibiotic stewardship: Physicians’ acceptance of AWaRe (Access, Watch, Reserve)-based microbiology reporting and its impact on judicious antibiotic selection – a cross-sectional study

**DOI:** 10.3205/dgkh000532

**Published:** 2025-03-04

**Authors:** Heera Hassan, Aravind Reghukumar, Jyothi Rajahamsan, Sreenadh Harikumar, Manjusree Shanmugham

**Affiliations:** 1 Department of Microbiology, Government Medical College, Thiruvananthapuram, Kerala, India; 2 Department of Infectious Diseases Government Medical College, Thiruvananthapuram, Kerala, India; 3 District Public Health Laboratory, Wayanad, Kerala, India: Sulthan Bathery, Wayanad, Kerala, India

**Keywords:** antimicrobial resistance, antimicrobial stewardship, AWaRe classification, nudging, reporting format

## Abstract

**Background:**

Inappropriate and excessive use of antimicrobials contributes to the rise of antimicrobial resistance (AMR). To address this, better tools are needed to improve antibiotic prescribing globally. This study evaluates a novel antimicrobial stewardship (AMS) tool – the AWaRe-based microbiology reporting format – developed by the Departments of Microbiology and Infectious Diseases at the Government Medical College, Trivandrum, Kerala state, India. This format incorporates the WHO AWaRe classification into bacterial culture susceptibility reports. The primary objective was to assess the effectiveness of this reporting format in encouraging physicians to choose access antibiotics. The secondary objective was to identify the most preferred microbiology reporting format for antimicrobial susceptibility reports.

**Materials and methods:**

A cross-sectional study was conducted among registered modern-medicine practitioners (physicians) in Kerala via social media platforms using a Google form-based questionnaire over a two-month period. Responses were automatically recorded and analysed using descriptive statistical methods. The study included all physicians who gave informed consent, excluding those who declined to participate. The sample size was determined to be 138 based on a pilot study.

**Results:**

A total of 228 physicians participated in the study, 137 (60.1%) of which were clinicians. Among the participants, 199 (87.2%) preferred the AWaRe-based reporting format over conventional formats for effective implementation of the Antimicrobial Stewardship Programme (AMSP). Using this AMS tool, 76.7% (n=56) of participants were successfully guided to choose appropriate access antibiotics.

**Conclusion:**

The study demonstrates that AWaRe-based susceptibility reporting formats can effectively nudge clinicians towards better antibiotic selection, supporting the successful implementation of AMSP (antibiotic stewardship programs). This format also serves as a continuous IEC (Information, Education and Communication) tool, helping clinicians familiarize themselves with WHO AWaRe classifications and encouraging the preferential selection of access-group antibiotics when appropriate.

## Zusammenfassung

**Hintergrund:**

Der unsachgemäße und übermäßige Einsatz von Antibiotika trägt zum Anstieg der Antibiotikaresistenz (AMR) bei. Um dieses Problem anzugehen, werden bessere Instrumente benötigt, um die Verschreibung von Antibiotika weltweit zu verbessern. In dieser Studie wird ein neuartiges Tool für den verantwortungsvollen Umgang mit Antibiotika (Antibiotic Stewardship, AMS) bewertet, das AWaRe-basierte mikrobiologische Berichtsformat, das von den Abteilungen für Mikrobiologie und Infektionskrankheiten am Govt Medical College, Trivandrum, entwickelt wurde. In diesem Format wird die AWaRe-Klassifizierung der WHO in die Berichte über die Empfindlichkeit von Bakterienkulturen integriert. Das primäre Ziel bestand darin, die Wirksamkeit dieses Berichtsformats zu bewerten, um Ärzte zu ermutigen, geeignete Antibiotika auszuwählen. Das sekundäre Ziel bestand darin, das bevorzugte mikrobiologische Berichtsformat für Bericht zur antibiotischen Empfindlichkeit zu ermitteln.

**Materialien und Methode:**

Es wurde eine Querschnittsstudie unter registrierten Ärzten für moderne Medizin in Kerala über Social-Media-Plattformen mit einem Google-basierten Fragebogen über zwei Monate durchgeführt. Die Antworten wurden automatisch aufgezeichnet und mit deskriptiven statistischen Methoden analysiert. In die Studie wurden alle Ärzte für moderne Medizin einbezogen, die ihre Zustimmung gaben. Ausgeschlossen wurden diejenigen, die die Teilnahme ablehnten. Die Stichprobengröße wurde auf der Grundlage einer Pilotstudie auf 138 festgelegt.

**Ergebnis:**

An der Studie nahmen 228 Ärzte teil, von denen 137 (60,1%) Kliniker waren. Von den Teilnehmern bevorzugten 199 (87,2%) das AWaRe-basierte Meldeformat gegenüber herkömmlichen Formaten für eine wirksame Umsetzung des Antibiotic Stewardship Programms (AMSP). Mit Hilfe des AMS-Tools wurden 76,7% (n=56) der Teilnehmer erfolgreich zur Auswahl geeigneter Antibiotika angeleitet.

**Schlussfolgerung:**

Die Studie zeigt, dass AWaRe-basierte Berichtsformate zur Antibiotikaempfindlichkeit Kliniker wirksam zu einer besseren Antibiotikaauswahl anregen können und die erfolgreiche Umsetzung des AMSP unterstützen. Das Berichtsformat dient auch als kontinuierliches IEC-Instrument, das den Klinikern hilft, sich mit den AWaRe-Klassifikationen der WHO vertraut zu machen, und die bevorzugte Auswahl von Antibiotika der Zugangsgruppe fördert, wenn das angebracht ist.

## Introduction

Antimicrobial resistance (AMR) is a threat to global health and development; each year it contributes to millions of deaths worldwide [1], [2]. Inappropriate use and overuse of antibiotics are contributing to the rise of antimicrobial resistance (AMR) and adversely affecting the efficacy of these vital medications [1]. AMSP (antimicrobial stewardship programs) have been implemented in healthcare settings to promote the appropriate and responsible use of antibiotics, with the aim of preserving their effectiveness for future[2] generations [2]. However, despite these efforts, the problem of antimicrobial resistance continues to escalate. There is a recognized need for functionally valuable tools to improve antibiotic prescribing globally [1]. In order to support antibiotic stewardship efforts at local, national and global levels, the WHO expert committee on selection and use of essential medicines in 2017 developed the AWaRe classification of antibiotics with the aim of emphasizing the importance of appropriate antibiotic selection [1]. AWaRe classification of antibiotics is based on their spectrum of activity and potential to induce as well as propagate resistance; it also identifies antibiotics that are priorities for monitoring and surveillance of use [3]. According to the WHO, to tackle AMR effectively, 60% of antibiotics prescribed should be from the access group by 2023 [1]; access antibiotics are first- or second-line treatments for common infections, and should be widely accessible]. It is considered a useful tool and was originally designed for monitoring antibiotic consumption [1], [4], [5] and the effects of stewardship policies that aim to optimize antibiotic use and curb antimicrobial resistance [6]. It is imperative that knowledge of the AWaRe tool be improved among physicians, as shown by many studies [7], [8], [9]. Therefore, novel teaching methods should be developed for a continuous learning experience. Furthermore, reporting formats have evolved over time, but with no evidence-based studies on how reporting of antibiotic susceptibilities should be done. As a solution to the above-mentioned problems, a new antibiotic stewardship nudging tool was devised jointly by the Departments of Microbiology and Infectious Diseases at the Government Medical College, Trivandrum, Kerala, India, by incorporating AWaRe-based classification into an antibiotic-susceptibility (by disc diffusion) reporting format. The aim of this tool was to create a continuous AMR reminder and to nudge doctors to select antibiotics from the access category wherever possible. Nudging in clinical microbiology is an AMS strategy that is used to influence decision making through strategic reporting of antimicrobial susceptibility results without compromising prescriber autonomy [10]. Previous studies have shown the effectiveness of nudge-based AMS [11], [12].

This study aimed to assess the effectiveness of the newly introduced AWaRe based reporting format as an AMS nudging tool, by serving as a continuous educator/reminder of AMR, which in turn would lead to better antibiotic prescription practices. The primary objective was to evaluate the effectiveness of AWaRe-based reporting in nudging physicians to choose access antibiotics. The secondary objective was to find the most preferred microbiology reporting format with respect to antimicrobial susceptibility reporting. The evidence gathered from this study may help in optimizing stewardship activities globally, especially in developing countries.

## Materials and methods

A cross-sectional study was conducted by the Department of Microbiology, Government Medical College, Thiruvananthapuram, Kerala, India among registered physicians actively engaged on social media platforms affiliated with professional bodies such as the Indian Medical Association (IMA), infectious disease physicians, and clinical microbiologists. The duration of study was a period of 2 months (August 13, 2023–October 13, 2024). 

A Google form-based questionnaire which also served as an IEC (Information, Education and Communication) tool on AWaRe classification was designed in the English language. This was circulated in social media to collect responses from willing participants. The questionnaire had 10 sections which assessed various aspects of knowledge, understanding and preferences on stewardship; it also ensured consent to participate in the study.

All physicians who gave informed consent were considered for inclusion in the study, while exclusion criteria comprised doctors who expressed unwillingness to participate. The minimum sample size was determined to be 138, based on the proportion of physicians preferring the AWaRe-based reporting format, as identified in a pilot study. The sample size calculation employed using the formula n=(Z_1–a/2_)2pq/d^2^ where p (proportion of physicians preferring the AWaRe-based reporting format)=90%, q=10%, d=5.

The study questionnaire was filled in by participants from across India. Responses were automatically recorded in Excel sheets and analysed using descriptive statistics, with results presented as frequencies and percentages. 

## Results

All study participants of the study were qualified modern-medicine physicians of various specialities who are eligible to prescribe antibiotics or validate susceptibility reports. 228 physicians participated in the study, as shown in Table 1 (Tab. 1).

**Figure 1:**
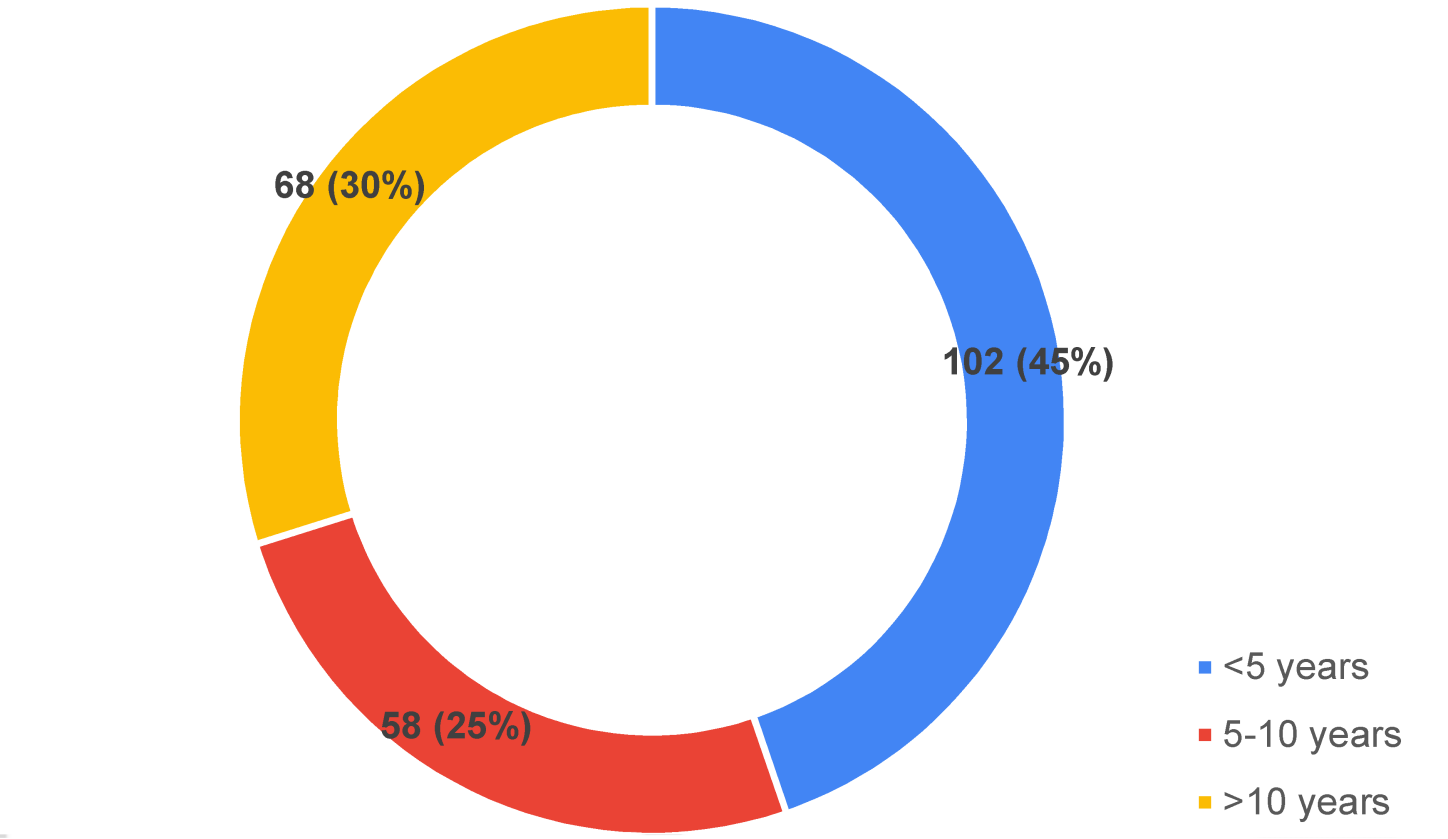
Participants based on years of clinical experience

**Figure 2:**
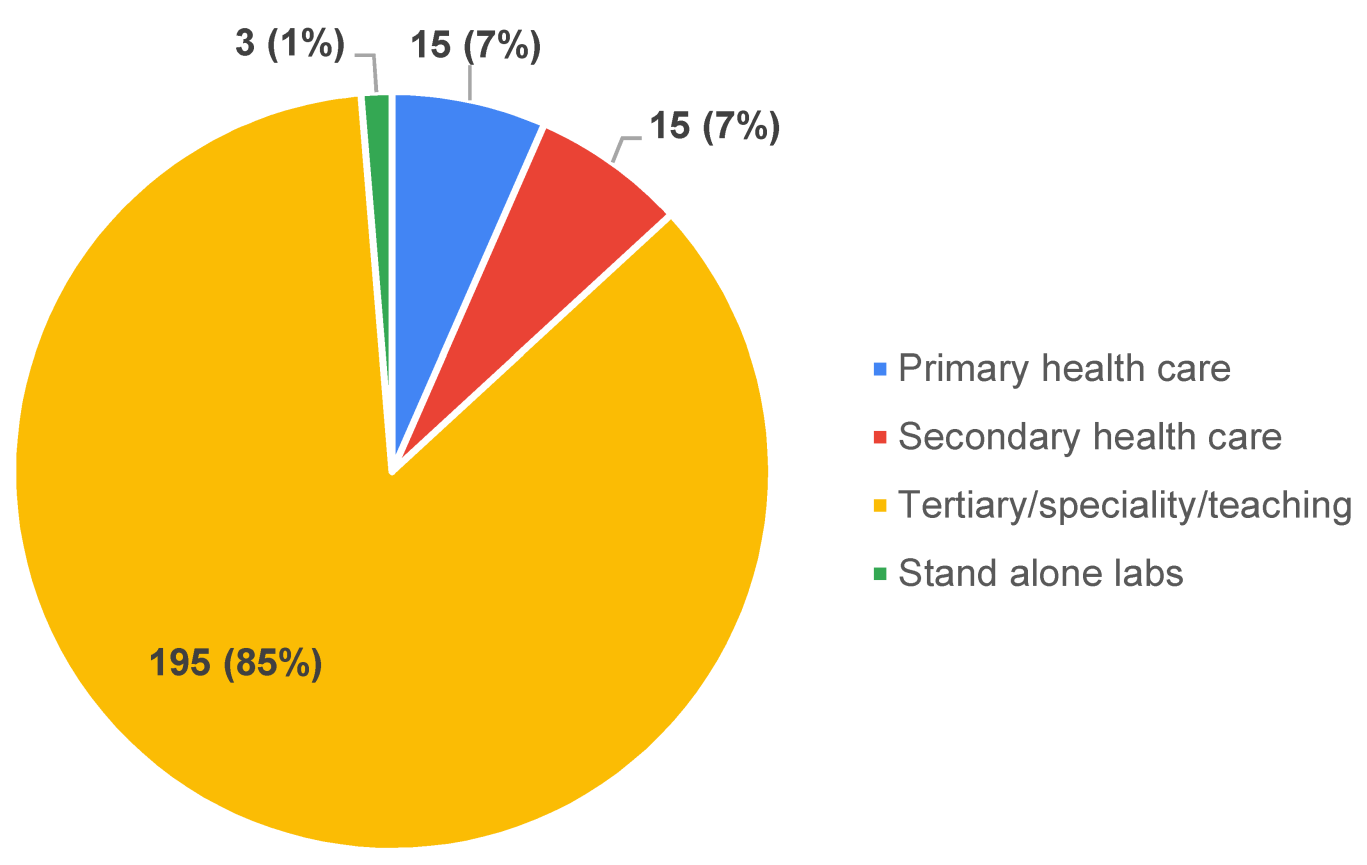
Institution-level classification of participants

Their experience in terms of years, associated with service and the level of health care with which they were affiliated, were also collected (Figure 1 (Fig. 1) and Figure 2 (Fig. 2)).

All participants demonstrated a comprehensive understanding of the critical role their prescription practices play in the development of antimicrobial resistance. Furthermore, when a definition was given and the participants were asked to identify which concept it described, 221 (97%) participants identified the definition as of antibiotic stewardship from the multiple options which were provided. Notably, 211 (92.5%) respondents either agreed or strongly agreed that the lack of awareness regarding proper antibiotic use is a contributing factor to the rising rates of resistance.

To assess participants’ knowledge regarding antibiotics and their efficacy against specific microorganisms, a question was posed comparing the effectiveness of vancomycin and cloxacillin in methicillin-sensitive *Staphylococcus aureus* (MSSA) treatment. Out of the study population, 117 (51%) correctly identified cloxacillin as more effective than vancomycin for MSSA treatment. However, 83 (37%) of participants, held the opposite belief, and 28 (12%) believed that the efficacy of both antibiotics was equal in treating MSSA. 

**Figure 3:**
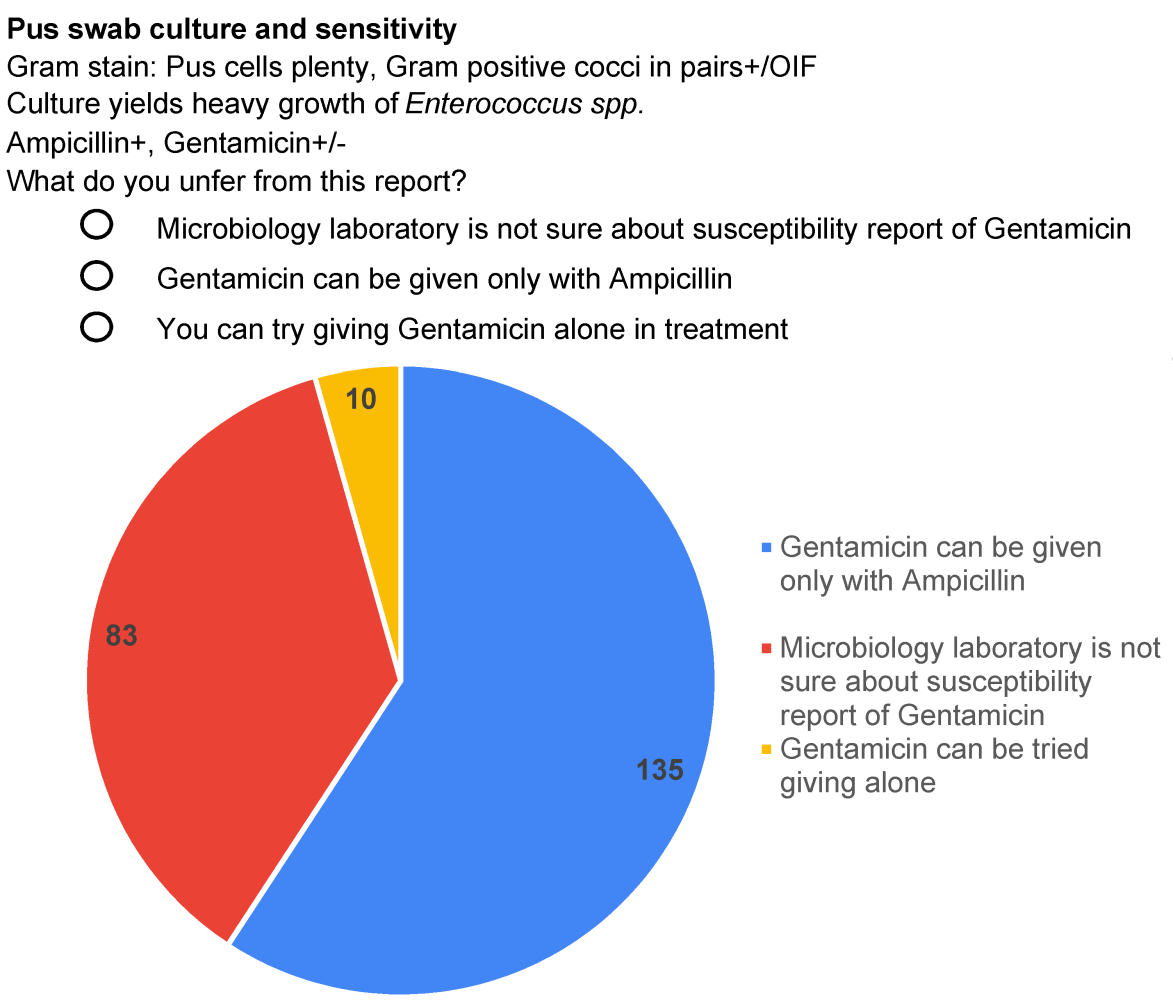
Question and interpretation on susceptibility reports of gentamicin synergy in culture reports of Enterococcus without proper comments

The study delved into the understanding of prescribers regarding susceptibility reports lacking comments. A classical positive-culture report of *Enterococcus* was presented, indicating the susceptibility report of ampicillin as + and synergistic activity of gentamicin as +/–, without any footnotes/ comments on what +/– indicated. The participants were asked to choose the right interpretation of the gentamicin report from a multiple choice answer panel; the choice of participants who opted for each option are shown in Figure 3 (Fig. 3). This question was based on the misconception of microbiologists that clinicians understand the meaning of reports without footnotes or comments. Notably, 83 (37%) of the study population perceived that the gentamicin +/– notation indicated uncertainty on the part of the microbiology laboratory regarding the gentamicin results as opposed to its synergy with ampicillin.

**Figure 4:**
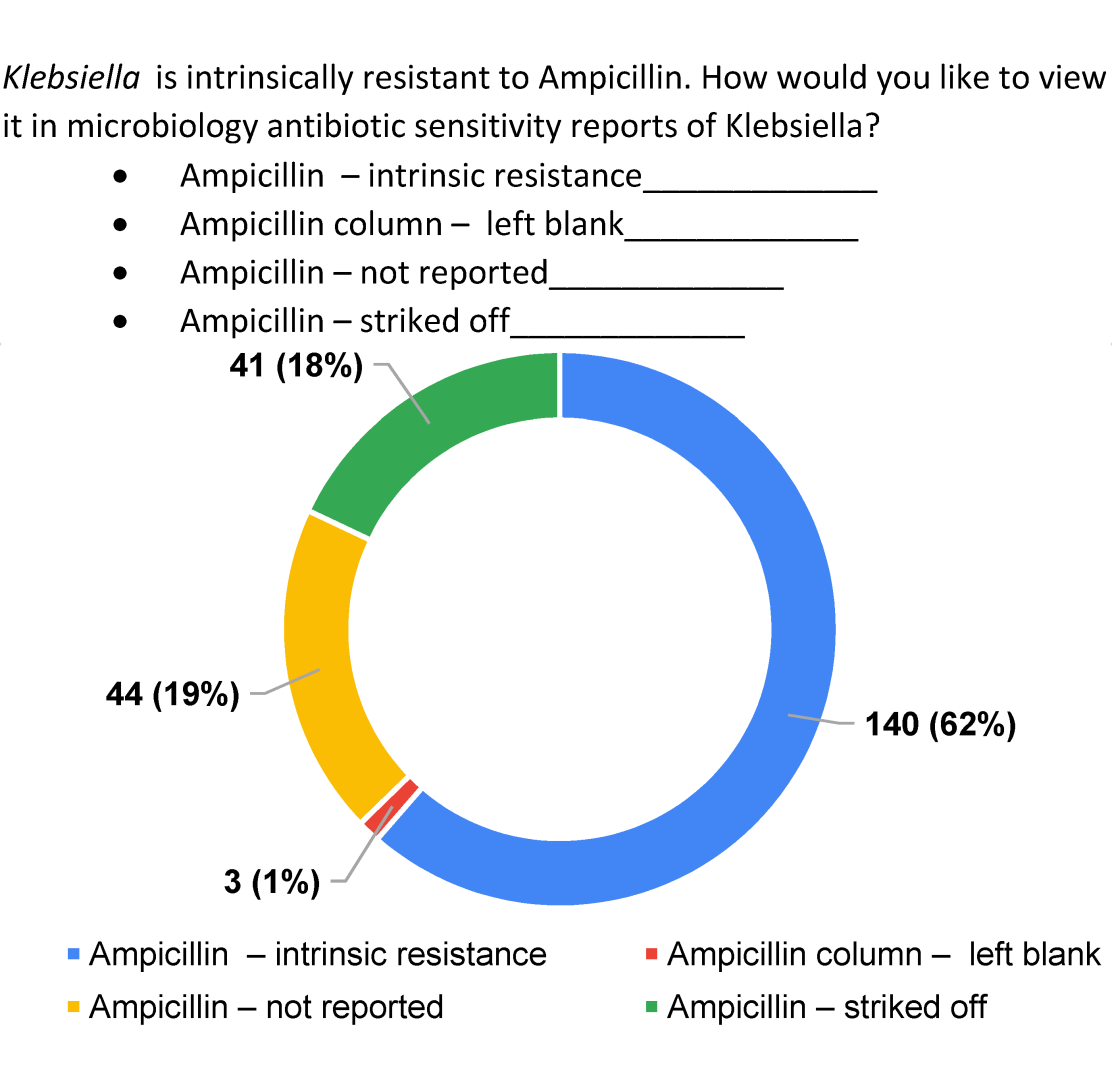
Preference question with options and preferences on reporting of resistance facts

The participants were queried about their preference for viewing intrinsic resistance reports of ampicillin in positive culture reports with *Klebsiella*. Predominant regional practices of reporting ampicillin for *Klebsiella* isolates were given as options. A substantial proportion – 62% (n=140) – of the study population expressed a preference for being educated about the intrinsic resistance aspect, diverging from the conventional “out of sight, out of mind” approach typically associated with selective reporting as shown in Figure 4 (Fig. 4). The study underscored the imperative need for ongoing information and education regarding intrinsic resistance. 

Familiarity with the AWaRe classification of WHO was determined by a question asking participants to choose the access antibiotics from a list. There were additional options for those uncertain about access antibiotics and those who had not heard of the AWaRe classification. Only 88 (38.5%) participants could correctly choose access antibiotics. 48 (21%) were not aware of such a classification. 

**Figure 5:**
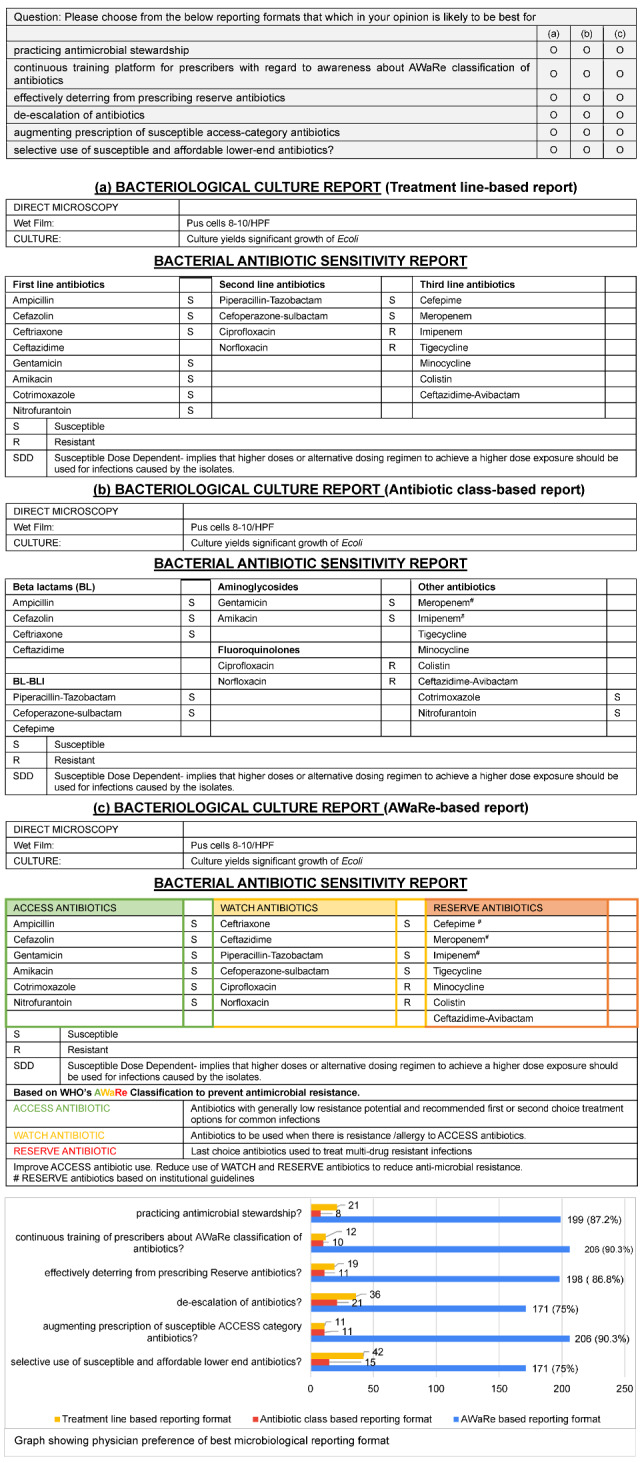
Question and preferences on the best microbiological reporting format

A comparative analysis was conducted between different formats – (a) treatment line-based, (b) antibiotic class-based and (c) AWaRe-based – of the same antibiotic susceptibility result of an *E. coli* isolate from a urine specimen. The study revealed a strong preference for the AWaRe-based reporting format as shown in Figure 5 (Fig. 5). Out of 228 participants , 206 (90%) participants were of the opinion that the AWaRe-based format augmented prescription of access-group antibiotics and also served as a continuous training platform for prescribers. About 171 (75%) participants felt that the AWaRe-based format helped in de-escalation of antibiotics and prescription of affordable antibiotics. About 199 (87.2%) participants thought that the same format helped in practicing antibiotic stewardship.

Sections 3 and 9 of the questionnaire had the same clinical scenario, with the same result, except in different susceptibility reporting formats, section 3 had a class-based format and section 9 had an AWaRe-based format. Out of the 73 participants who initially could not identify the correct access antibiotic in section 3 of the questionnaire, 56 successfully did so at the end of the study in section 9 when presented with the AWaRe-based format. This demonstrated an effectiveness of 76.7% for the AWaRe-based reporting format as an AMS nudging tool.

## Discussion

Antimicrobial resistance (AMR) is one of the major public health problems of the 21^st^ century that threatens the effective prevention and treatment of infectious diseases that no longer respond to the common medicines used to treat them. Since the discovery of penicillin, bacteria have developed resistance to every new antibiotic introduced. Therefore, it is crucial to take action to prevent a looming global healthcare crisis [13]. It is estimated that 10 million deaths annually may be attributable to AMR by 2050, with a predicted global economic cost of 100 trillion USD. This has prompted the World Health Organization (WHO) to identify antimicrobial resistance (AMR) as a threat to global public health [14], emphasizing on the need for urgent multisectoral action to slow it down [15]. One of the factors causing the rise in AMR rates is the overuse and misuse of antibiotics. A desire for high cure rates, knowledge gaps on the advantages of de-escalation, as well as ignorance on antibiotic choice are the major drivers of antibiotic overuse. Stewardship activities include a set of actions which promote judicious use of antimicrobials to ensure access to therapy for all who need it, even for the future [16]. Despite the existence of many stewardship tools, there has been an increasing demand for newer and more effective tools for AMSP which can educate and persuade doctors. 

Compared to a previous study [9] where only 62% of the prescribes knew the definition of AMSP, this study showed 97% knowledge of its definition. All the participants were fully aware of the importance of their prescription practices in the development of antimicrobial resistance. 

In a previous study by Rahbi et al. [7], 49% agreed that widespread use of antibiotics contributed to AMR. In our study, 92.5% of the participants either agreed or strongly agreed that it is the lack of awareness on proper antibiotic use which contributes to increasing rates of resistance. Further questions were designed to elucidate whether the study population knew that vancomycin was less effective than cloxacillin in the treatment of MSSA. About 12% believed that they were equal. About 37% were not aware of the superiority of cloxacillin over vancomycin in treatment of MSSA. This raises concerns about misuse of vancomycin for MSSA, hinting at the imminent risk of resistance to vancomycin in the near future. These facts emphasize the importance of implementing customized IEC activities as a part of AMSP . 

The section of the study on participants’ comprehension of the susceptibility reports without proper comments showed that 41% of the study population were completely unaware of the synergistic activity of gentamicin with ampicillin in treating *Enterococcus*, out of which 4% would even prescribe gentamicin alone for such patients. This makes it imperative for clinical microbiology reports to be equipped with self-explanatory footnotes and comments to guide rational antibiotic treatment. To add to the importance of reporting formats serving as a communication medium with correct interpretations, 62% of the doctors preferred to be educated on intrinsic resistance of different antibiotic combinations through microbiology susceptibility reporting formats. In 2020, Deepasree et al. [17] emphasized the need for a compulsory requirement to provide adequate comments as footnotes in the susceptibility reporting formats to help clinicians rationalise and improve their antimicrobial prescription practices. Hence, microbiology reporting formats could serve as valid educational/informative tools of AMSP to guide proper use of antibiotics by often-overworked clinicians. This is particularly important in developing countries where the physician-to-patient ratio is very low, and doctors struggle to keep up with the workload and challenges of advancing healthcare.

Despite the World Health Organization (WHO) introducing the AWaRe classification in 2017 as a measure to optimize antibiotic use, its utilization has been confined primarily to pharmacists supporting stewardship activities. The limited awareness or information among physicians regarding the resistance potential of various antibiotics and the AWaRe classification poses a risk of overusing antibiotics with high resistance potential, particularly those classified under the “watch” and “reserve” categories. In this study, only 38.5% of the participants were aware of the AWaRe classification and could correctly choose the access antibiotics. A previous study in Northern India [18] showed that only 33.3% of participants were aware of the AWaRe classification. The rates in both that and our study are low and comparable. This makes it highly important that such interventions of public health significance are given adequate emphasis and widespread publicity. 

In the absence of previous studies on the comparison of microbiology susceptibility reporting formats, our study stands out as the first study of its kind on the comparison of different microbiology formats for AMSP. Of the 228 participants, 90% of the study population suggested that AWaRe-based susceptibility reporting formats augmented prescription of access antibiotics and served as an educational medium for antibiotic updates. Additionally, with this reporting format, 76.7% of study participants (who were unable to choose access antibiotics with conventional reporting formats) could be rightly guided to choose access antibiotics.

## Conclusions

This study clearly shows that AWaRe-based microbiology susceptibility reporting formats are very effective AMS nudging tools and can serve as a continuous IEC platform to educate prescribers on AMSP, compared to conventional microbiology reporting formats based on antibiotic classes and treatment lines. Widespread use of this format might facilitate the preferential prescription of antibiotics from the access category, which in turn will help in attaining the WHO target of having 60% of prescriptions be from the access category.

### Limitations of the study

This study was based in a tertiary care teaching hospital, so that 85% of the participants were in fact from tertiary/teaching/speciality hospitals. The number of responses from primary and secondary care institutions were low.

Studies comparing reporting formats were not found despite extensive literature review. Hence, this study stands alone as the first study of its kind and has no comparison.

## Notes

### Competing interests

The authors declare that they have no competing interests.

### Ethical approval 

Ethical approval was obtained from the Institutional Ethics and Research Committee HEC NO:13/08/2023/MCT dated 05/09/2023 and according to Helsinki Declaration of 1975 (revised in 2013).

### Funding

None. 

### Acknowledgments

We gratefully acknowledge the excellent participation of all the doctors who helped us complete the study.

### Authors’ ORCID

- Hassan H: https://orcid.org/0009-0008-7090-372X
- Reghukumar A: https://orcid.org/0000-0002-5332-0978
- Jyothi R: https://orcid.org/0009-0000-0418-1265
- Harikumar S: https://orcid.org/0009-0006-2342-6751
- Manjusree S: https://orcid.org/0009-0000-5314-716X

## Figures and Tables

**Table 1:**
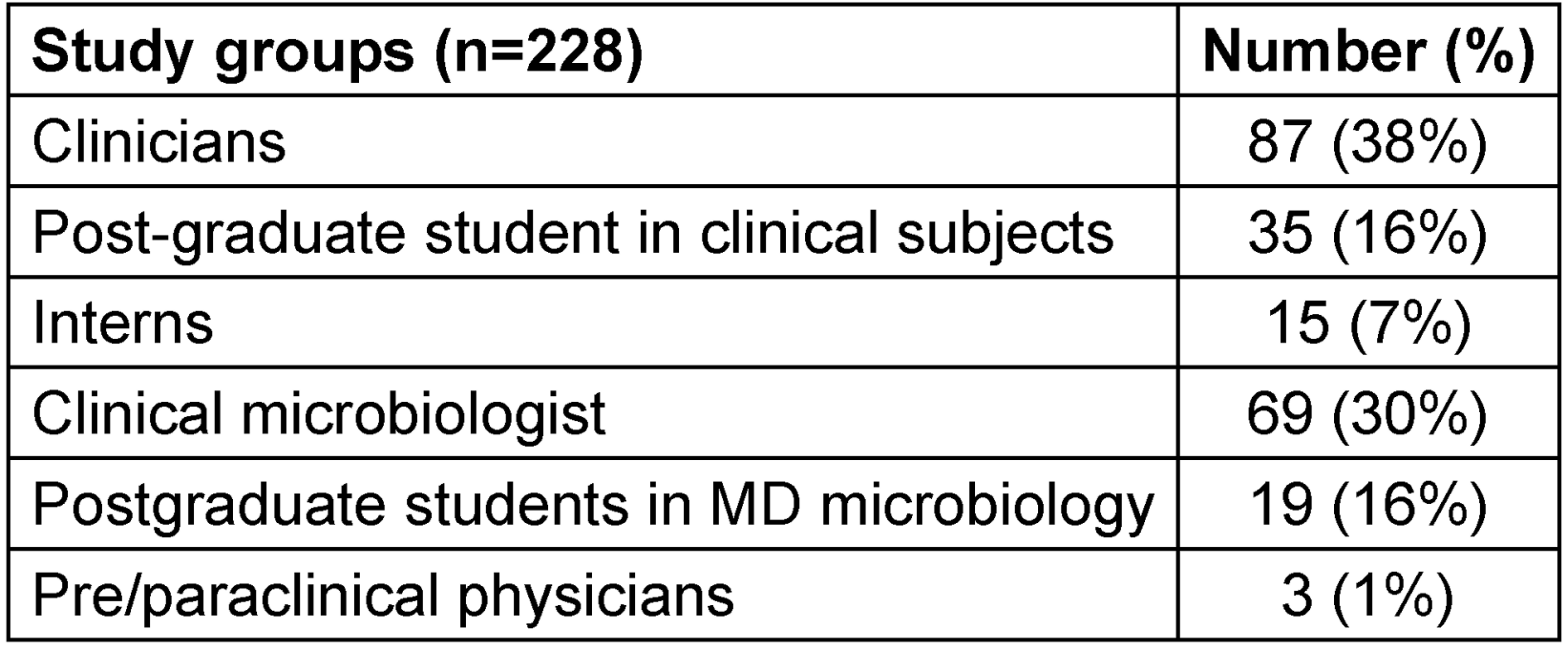
Study participants
